# Content Analysis of Work Limitation, Stanford Presenteeism, and Work Instability Questionnaires Using International Classification of Functioning, Disability, and Health and Item Perspective Framework 

**DOI:** 10.1155/2013/614825

**Published:** 2013-12-28

**Authors:** Vanitha Arumugam, Joy C. MacDermid, Ruby Grewal

**Affiliations:** ^1^University of Western Ontario, Health and Rehabilitation Sciences, 1201 Western Road, London, ON, Canada N6G 1H1; ^2^Rehabilitation Science, McMaster University, School of Rehabilitation Science, Hamilton, ON, Canada L8S 4L8; ^3^Clinical Research, Hand and Upper Limb Center, St. Joseph's Hospital, 268 Grosvenor Street, London, ON, Canada N6A 4L6

## Abstract

*Background*. Presenteeism refers to reduced performance or productivity while at work due to health reasons. WLQ-26, SPS-6, and RA-WIS are the commonly used self-report presenteeism questionnaires. These questionnaires have acceptable psychometric properties but have not been subject to structured content analysis that would define their conceptual basis. *Objective*. To describe the conceptual basis of the three questionnaires using ICF and IPF and then compare the distribution and content of codes to those on the vocational rehabilitation core set. *Methods*. Two researchers independently linked the items of the WLQ-26, SPS-6, and RA-WIS to the ICF and IPF following the established linking rules. The percentage agreement on coding was calculated between the researchers. *Results*. WLQ-26 was linked to 62 ICF codes, SPS-6 was linked to 17 ICF codes, and RA-WIS was linked to 74 ICF codes. Most of these codes belonged to the activity and participation domains. All the concepts were classified by the IPF, and the most were rational appraisals within the social domain. Only 12% of codes of the core set for vocational rehabilitation were used in this study to code these questionnaires. *Conclusion*. The specific nature of work disability that was included in these three questionnaires was difficult to explain using ICF since many aspects of content were not confined. The core set for vocational rehabilitation covered very limited content of the WLQ-26, SPS-6, and RA-WIS.

## 1. Introduction

Rehabilitation is based on an understanding that health and function extend beyond the presence or absence of disease to include the ability to participate in life activities and roles. Similarly, we now recognize that work functioning extends beyond the presence or absence of being at work to include the ability to engage in work activities and roles. Presenteeism refers to reduced performance or productivity while at work due to health reasons [1]. In a study conducted in Sweden where one-third of the surveyed labor force reported going to work two or more times in the past year in spite of their health being so bad that they should have taken leave [2]. Presenteeism is a complex issue that is affected by individual, work, workplace factors, health, and health behaviours. Previous studies have tried to identify determinants of presenteeism and have identified factors like low monthly income, psychological stress, initial health, time pressure, and finding a replacement, amongst others [1–8].

During rehabilitation, ability to return to work is often a major concern. Vocational rehabilitation is a specific subtype of rehabilitation that focuses on helping those with disabilities to regain skills and abilities that allow them to acquire or retain employment. It is important to have questionnaires that allow one to quantify the amount of difficulty experienced at work to monitor the success of these rehabilitative processes.

People can return to work for a variety of reasons such as financial responsibilities or social responsibility to coworkers. Thus, return to work cannot be the only indicator of successful outcome of vocational rehabilitation. Presenteeism is responsible for a substantial burden to the employee and the employer in lost productivity. The economic burden caused due to sickness presenteeism is attributable to work impairment, disability, and lost productivity time. [9] Studies that identified the economic burden of sickness presenteeism due to depression found that the direct and indirect costs exceeded 18.2 billion in USA and 15.1 billion in UK [10].

The three presenteeism questionnaires that have the most supporting psychometric evidence are (1) 26-Item Work Limitations Questionnaire (WLQ-26) [11], (2) Stanford Presenteeism Scale (SPS-6) [12], and (3) Rheumatoid Arthritis Work Instability Scale (RA-WIS) [13]. The recent systematic review of the psychometric properties of these questionnaires revealed that all three have been assessed in various populations and have demonstrated acceptable levels of validity, reliability, and responsiveness [14].

The International Classification of Functioning Disability and Health (ICF) is an international classification developed by the World Health Organization (WHO) [15]. The domains of the ICF are classified into body structures, body functions, activity limitations, and participation restrictions. There are also contextual factors that are taken into account, which include individual environmental factors and personal factors although the latter are not classified [15]. ICF provides a theoretical model that underpins a substantial component of rehabilitation research. It also provides a classification system and language to help communicate about disability. Processes has been developed to link content from questionnaires to this hierarchical coding system Cieza et al. [16].

Core set for vocational rehabilitation [17] has been developed by a rigorous multistep process, for example, engaging stakeholders through international consensus, informed by qualitative and quantitative research findings with the goal of establishing the most relevant codes for specific areas of practice or health problems. There is a comprehensive and a brief core set to provide options across diverse applications. The comprehensive set has ninety codes while the brief core set has thirteen codes. These core set were established to describe the functioning and participation of individuals, for instance, those who can participate in multidisciplinary vocational rehabilitation. Since vocational rehabilitation aims at successful return-to-work, participating in all life activities, sports, and so forth presenteeism questionnaires may be highly relevant to the outcome of research and practice in this area. Hence, it is necessary to code the concepts of the presenteeism questionnaires with the codes of ICF which would ensure comparability and interpretability of the presenteeism questionnaire scores across studies, transcending language, and cultural barriers [18, 19].

The Item Perspective Framework (IPF) is a classification system developed to classify the content of individual items of questionnaires as to what kind of decisions respondents will have to make in responding to a question [20]. IPF was derived from the philosophical work on how individuals appraise “value” or “quality” in life by Pirsig [21] and McWatt [22]. The IPF was constructed to classify fundamental qualities of the items in a patient report outcome measure such as (1) the type of appraisal presented (rational or Emotional), (2) the nature and form of concepts under evaluation, and (3) the types of relationships that occur among multiple concepts [20]. The developer has proposed a novel way of using “item perspective” in conjunction with the ICF classification [20], The ICF is concerned with an aspect of addressed functioning, but does not code the perspective [23] nor does it have a mechanism to address the relationship between different concepts [24]. These two are important issues for understanding the content of questionnaires and are included in the “items perspective” classification. The IPF can provide this information making the content analysis more meaningful.

Content validation can be enhanced by using rigorous methods of evaluating content. This can be particularly significant for complex concepts like presenteeism. The purposes of the current study are threefold: (1) to link the three presenteeism questionnaires to the ICF, (2) to use the IPF framework in conjunction with the ICF classification to classify an item as emotional, rational, and also categorize them into biological or inorganic or psychological or social domain, and (3) to compare the distribution or content of codes included in these questionnaires to those on the vocational rehabilitation core set.

## 2. Methodology

### 2.1. Instrument Description

#### 2.1.1. 26-Item Work Limitations Questionnaire (WLQ-26)

The Work Limitations Questionnaire exists in various formats including a 26 item version [11] and an 8-item version [25]. The 26-item version was used in the study. The main purpose of this questionnaire is to measure the impact of chronic health problems and or treatment on a person's perceived ability to handle work demands [11]. It also measures health-related productivity loss. It includes 26-item under 4 subscales addressing four dimensions of job demands: it uses a 5 point ordinal response scale with an additional sixth option. The physical demand sub-scale contains six items covering physical demands, energy drive, moving from place to place, flexibility tasks and coordination of the hand “does not apply to my job.” The time demand subscale contains five items from managing, scheduling, and completing a job. The mental/interpersonal demands contain nine items that assess cognition and on the job social interactions. The output demand subscale contains six items determining the quality and productivity at work. The total scores range from 0 to 100% [26].

The WLQ has been validated with a variety of patient population with chronic conditions such as rheumatoid arthritis (RA), depression, osteoarthritis, back pain, migraine, and epilepsy [11, 27]. It is shown to have high internal consistency with Cronbach's alpha values ranging from 0.88 to 0.97 [11, 27]. In the initial validation study, the interclass correlation coefficient (ICC) for specialty clinic patients for 2 week test-retest validity ranged from 0.69 to 0.80 for the 4 sub scales [9]. The WLQ has shown good construct validity by correlating significantly with arthritis, pain, stiffness, functional limitation, and self-reported work productivity [27]. Reliability coefficients are reported to range from 0.70 to 0.90 for all items and from 0.88 to 0.91 for items within each scale.

#### 2.1.2. Stanford Presenteeism Scale (SPS-6)

SPS-6 is a 6-item self-report questionnaire [12]. It measures the impact of a worker's perceived ability to concentrate on work tasks despite the distractions of health impairments and pain. The response scale for the SPS-6 is a 5-item Likert scale, with response options ranging from 1—strongly agree to 5—strongly disagree, giving a total score that could range between 6 and 30. A total score would not be calculated if response to any of the items was missing.

SPS-6 has exhibited acceptable levels of validity and reliability [28]. In terms of construct validity SPS-6 exhibited mild to moderate correlation with other work productivity questionnaires. (Spearman's Rho 0.41–0.69) [28] It has an internal consistency with Cronbach's alpha = 0.71 [28].

#### 2.1.3. Rheumatoid Arthritis Work Instability Scale (RA-WIS)

The RA-WIS is a 23 item self-report questionnaire that assesses work instability. Work instability is the consequence of a mismatch between an individual's functional ability and his/her work tasks that place the individual at risk for work disability (lowered productivity/premature job loss, etc.) [13]. It has no subscales, and it has a dichotomous response option of yes or no only. The total score ranges from 0 to 23. The WIS can be subgrouped into 3 bands indicating low (less than 10), medium (10–17), and high (above 17) risk of work disability.

The RA-WIS was specifically developed for patients with rheumatoid arthritis [13] but it has been validated for use in other groups of diseases such as osteoarthritis (OA) [29, 30]. It has excellent test-retest reliability of 0.89 (Spearman's rho) [13]. Beaton et al. have found that the RA-WIS exhibits excellent correlations with other presenteeism questionnaires [28]. In workers with OA, it exhibited moderate to high correlations. (*r* = 0.55–0.79) [31]. It is also found to have predictive validity (relative risk = 1.05). In terms of responsiveness, RA-WIS has shown to exhibit small to moderate standardized response means (SRM) and effect size (ES) [28].

### 2.2. Procedure

Two academic physical therapists highly qualified and trained in the field of ICF coding independently linked the items of the WLQ-26, the SPS-6, and the RA-WIS to the ICF and IPF. Percentage agreement (*A*) was calculated between the raters for the linking process.

#### 2.2.1. ICF Linking

The ICF linking procedures were carried out following the eight standardized linking rules proposed by Cieza et al. [16, 19]. As per these linking rules, meaningful concepts should be identified from the items of the questionnaire and the identified concepts must be linked to the most precise ICF category. Items with insufficient information about the meaningful concepts, about the precise ICF category to which it should be linked, are to be marked as “nd = not definable.” Meaningful concepts that are related to health, physical health or mental (emotional) health in general, are assigned “nd-gh” (not definable-general health), “nd-ph” (not definable-physical health) or “nd-mh” (not definable-mental health), respectively. Meaningful concepts related to quality of life are assigned “nd-qol” (not definable-quality of life). When the meaningful concept is not covered with the codes of the ICF, and if it is not a personal factor, then the meaningful concept is assigned “n/c = not covered.” If the meaningful concept refers to a diagnosis or health condition, the meaningful concept will be assigned “hc” (health condition) [16]. Code d 840–859 deals with work and employment. Since all the items of all three questionnaires deal with work and employment, we have used these codes as mandatory codes to code all items of the three questionnaires.

#### 2.2.2. Classification Using IPF

The items were classified based on the guidelines proposed by Rosa [20]. The first three steps of the proposed five step process were used in this study. In the first step, the context of the item, that is, the declared purpose of the questionnaire, was determined. In the second step the type of appraisal presented with the item (rational or emotional) was determined. Items were classified as presenting “emotional” appraisals (E) only when they assess the respondent's emotions or feelings at the present time. Any inquiries into emotions/feelings that have occurred in the *past* or “*in general*” are classified as rational appraisals (R) since they require retrieval of memories pertaining to previous psychological states. In the third step, the concept domains represented in the item are identified. According to the IPF framework there are four concept domains that represent all subjective and objective evolutionary levels of reality that are amenable to human perception namely inorganic (I), for example, does your chair has a proper arm rest?, biological (B), for example, “rate the level of your shoulder pain?,” social (S), for example, “has pain affected your social life?” or psychological (P), “are you depressed and you feel less capable because of your shoulder pain?” [20] These concept domains have a hierarchical order reflecting McWatt's hypothesis that the inorganic matter gives rise to (and supports) biological organisms; biological organisms self-organize and interact with one another in a manner that gives rise to social behaviors and psychological functioning occurs as a result of increasing complexity in social behavior [22].

### 2.3. Analysis

Percentage agreement (*A*) between the raters was calculated by dividing the observed agreement (*O*) by the possible agreement (*P*). That is, *A* = (*O*/*P*) ∗ 100 [32].

To compare the distribution/content of codes in these questionnaires to those on vocational rehabilitation core set we used the following indicators:

(1) Alignment with core set (questionnaire to brief or comprehensive core set absolute linkage): It is the percentage of items from a questionnaire that could be linked to ICF core set codes.
  (1) =Questionnaire  items  that  are  linked  to  codes  appearing  in  the  core  setTotal  number  of  items  on⁡  the  measure  ×100%.

(2) Scope (Core Set Representation): It is the percentage of core set codes that appear when the measure's items are linked to ICF codes. This represents the extent to which the entire scope of content defined by the Core Set is represented on the measure/measure.

(2) =Number  of  questionnaire  items  that  are  linked  to  the  Core  SetTotal  number  of  codes  on⁡  the  (Brief  or  Comprehensive)  Core  Set.

Radar plots were used to describe the percentage of questionnaire items that fell under each of the domains of the ICF and IPF. Similarities and differences between the questionnaires as to the domains into which the items fall and a percentage of ICF codes or IPF codes used were identified. Content overlap between the questionnaires was also identified using these plots.

## 3. Results

### 3.1. WLQ-26

#### 3.1.1. Linking to ICF

**Figure 1:**
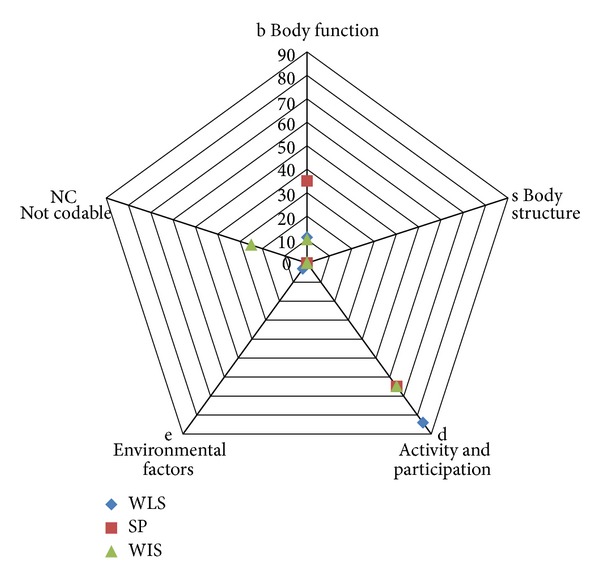
Radar plot showing the distribution of the items of the three presenteeism questionnaires between the domains of ICF. WLQ-26: The 26-item Work Limitation questionnaire; SPS-6: Stanford Presenteeism Scale-6; RA-WISR: heumatoid Arthritis- Work Instability Scale.

The 26 items were linked to 70 ICF codes, “of which 7 codes belong to the ICF body function; b codes; 3 codes belong to ICF environmental factors e codes, 60 belong to ICF “activity and participation.” (see Figure 1 and Table 1). Some of the observations are: there were 2 items in the questionnaire (items 15 and 16) that used 3 codes. 3 Items (17, 18, and part of 19) had the same codes (d230, d2301, and b140). Item 9 was not definable (“nd”) as ICF did not have any codes to code the meaningful concept of “sense of accomplishment”.

#### 3.1.2. Classification Using IPF

37 (90.2%) concepts required rational appraisals and 4 (9.8%) required emotional appraisals. 26 (63.4%) concepts fell within the social domain, 3 (7.3%) within the biological domain, 6 (14.6%) within the psychological domain, and 6 (14.6%) within the inorganic domain.

#### 3.1.3. Percentage Agreement

The between-rater agreement for the ICF linking was 84% and 100% for the IPF.

Distribution of codes in WLQ-26 to those in vocational rehabilitation core set as follows. The WLQ-26 was linked to 70 ICF codes. The alignment of the questionnaire was 100% with the mandatory work (d 840–859) codes being used. However, the alignment came down to 7% (brief core set) and 88% (comprehensive core set) when the mandatory codes were not considered (see Table 4). In terms of scope, 20 (28.6%), codes appeared in the comprehensive core set for vocational rehabilitation. Only 5 (7.1%) codes from the brief core set for vocational rehabilitation were used to code the concepts in WLQ-26 (see Table 4).

### 3.2. SPS-6

#### 3.2.1. Linking to ICF

The 6 items of SPS-6 fell equally under the body function and participation domain as indicated by the radar plot (see Figure 1). The 6 items on the questionnaire were linked to 17 ICF codes from 2 chapters, of which 6 codes belong to the ICF body function, b codes, 11 codes belong to ICF activity and participation, d codes (see Table 2). There were no meaningful concepts that referred to body structures and environmental factors.

#### 3.2.2. Classification Based on IPF

Four (30.8%) concepts required rational appraisals and 9 (69.2%) required emotional appraisals. Six (46.2%) concepts fell within the social domain, 4 (30.8%) within the biological domain, and 3 (23.1%) within the psychological domain. There were no concepts that fell within the inorganic domain.

#### 3.2.3. Percentage Agreement

While coding SPS-6 both raters agreed at all instances giving a between-rater percentage agreement of 100% for both the ICF linking and the IPF classification.

#### 3.2.4. Distribution of Codes in SPS-6 to Those in Vocational Rehabilitation Core Set

The SPS-6 was linked to 11 ICF codes. The alignment of the questionnaire was 100% with the mandatory work codes being used. The alignment came down slightly to 83% (brief core set) and it remained the same in the comprehensive core set when the mandatory codes were not considered. (see Table 4) In terms of the questionnaire, 6 (54.5%) codes appeared in the comprehensive core set for vocational rehabilitation. Only 2 (18.2%) codes from the brief core set for vocational rehabilitation were used to code the concepts in SPS-6 (see Table 4).

### 3.3. RA-WIS

#### 3.3.1. Linking to ICF

The meaningful concepts of the 23 items of this questionnaire were linked to 74 ICF codes from 6 chapters, of which 8 codes belong to the ICF body function (b codes), 48 codes belong to ICF activity and participation, d codes (see Table 3).

One or more meaningful concepts from the 18 items could not be coded by using ICF; the first part was codable whereas the second part was not codable (nc). The code d-850 Work and employment is used to code all the items in this questionnaire. There were concepts that the ICF could not capture in this particular questionnaire because a number of items address to emotional issues related to work. ICF considers emotional control as a body function but does not consider emotional perspectives.

#### 3.3.2. Classification Based on IPF

**Figure 2:**
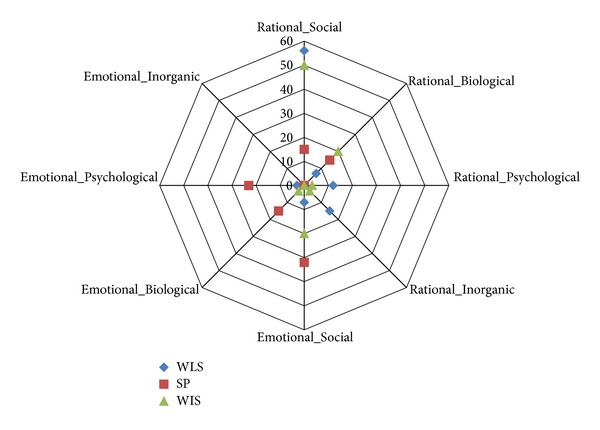
Radar plot showing the classification of the items of the three presenteeism questionnaires based on IPF. WLQ-26: The 26-item Work Limitation questionnaire; SPS-6: Stanford Presenteeism Scale-6; RA-WIS: Rheumatoid Arthritis-Work Instability Scale.

The item context and type of appraisal were determined revealing that 23 (76.7%) concepts required rational appraisals and 7 (23.3%) required emotional appraisals. 21(70%) concepts fell within the social domain, 7 (23.3%) within the biological domain, 1 (3.3%) within the psychological domain, and 1 (3.3%) within the inorganic domain (see Figure 2).

#### 3.3.3. Percentage Agreement

The between-rater percentage agreement for both ICF linking and IPF classification was 100%.

### 3.4. Distribution of Codes in RA-WIS to Those in Vocational Rehabilitation Core Set

The RA-WIS was linked to 35 ICF codes. The alignment of the questionnaire was 100% with the mandatory work codes being used. However, the alignment came down to 9% (brief core set) and 30% (comprehensive core set) when the mandatory codes were not considered (see Table 4). With regards to the scope of the questionnaire, 8 (22.9%) of the RA-WIS codes appeared on the comprehensive core set for vocational rehabilitation. Only 4 (11.4%) codes from the brief core set for vocational rehabilitation were used to code the concepts in RA-WIS (see Table 4).

### 3.5. Dimensionality and Content Overlap of the Three Presenteeism Questionnaires

#### 3.5.1. ICF

In terms of dimensionality, more than 80% of the items of the WLQ-26 and more than 60% of the SPS-6 and the RA-WIS items fell within the activity and participation domain (see Figure 1), while more than 30% of SPS-6 items and around 10% of the items of the WLQ-26 and RA-WIS fell within the body function domain. There were very minimal items from all three questionnaires that fell in the body structure or environmental domains. In summary, all three questionnaires focused on activity and participation in demonstrated overlapping content, but the SPS-6 is substantial emphasis on body function.

#### 3.5.2. IPF

Nearly all of the items of the WLQ-26 and the RA-WIS classified as rational appraisals. (See Figure 2) of which more than 50% of the items from both the questionnaires fell within Rational_Social domain; around 10% of items from both questionnaires fell under Rational_Biological domain. Around 15% of the items from the WLQ-26 fell under Rational_Psychological and Rational_Inorganic domains each while 20% of the items of the RA-WIS fell under the Emotional_Social domain. For SPS-6 around 70% of items were classified as requiring emotional appraisals while 30% required rational appraisals.

## 4. Discussion

By linking the items of the most commonly used presenteeism questionnaires, 26-Items Work Limitations Questionnaire (WLQ-26); Stanford Presenteeism Scale (SPS-6) and Rheumatoid Arthritis Work Instability Scale (RA-WIS) to the ICF language codes, we have shown that there is, substantial variability in the content that each address. Further, classification using the IPF reveals that the items of these three questionnaires contain many emotional appraisals that the ICF would not normally capture. We also found that many of the codes used to classify the presenteeism questionnaires were not reflected on the core set for vocational rehabilitation. Similarly, many codes from the core set were not reflected on the questionnaires.

The results of the current study show that none of the three questionnaires covered all the four domains of the ICF conceptual framework. The environmental domain is important and work since it includes many factors that would influence successful return to work including labor and workplace policies, and social environment. Other questionnaires have been developed to assess workplace and practice policies [1] and given the scope of environmental issues that can affect work it may be appropriate that environment is addressed in entirely separate questionnaires. More than fifty percent of the items in the three questionnaires are linked to the participation restriction domain of ICF. A small percentage (less than ten percent) of concepts are mapped onto body functions and environmental factors domain and none of the concepts mapped on to the body structure domain. These findings are consistent with the conceptual basis of presenteeism or at work disability since it focuses on a specific form of participation.

Our results indicate that these questionnaires tap into a limited number of concepts endorsed by the WHO when establishing the vocational rehabilitation core set. The WLQ-26 scale provided the most coverage of core set codes. It is not reasonable to expect a questionnaire to addresses all possible components of the construct it measures, but an adequate sampling of the key disability concepts should be present on the disability questionnaire that is targeted to the same conceptual basis. Whether a brief measure like the SPS-6 adequately addresses the scope of presenteeism is not clear. However, attention should be paid to what elements of the concept are covered and how they match the context when questionnaires are relied upon to evaluate the impacts of health problems or the benefits of interventions. It may be necessary to change or supplement questions to achieve an appropriate alignment between the context for measuring and the tools selected, particularly for vocational rehabilitation.

All three questionnaires had items that were linked to multiple meaningful concepts. For example, item 15 of WLQ-26 “Sit, stand, or stay in one position for longer than 15 minutes while working,” item 16 of WLQ-26 “bend, twist, or reach while working,” each have 3 different tasks. Respondents would have variable levels of difficulty with these tasks. Having multiple concepts within a single item on a questionnaire can be a way to bring together a group of activities or tasks that have a common etiological link to specific disorders. This can improve the efficiency of measuring difficulty since it may tap into different manifestations of a particular problem. However, in other cases this can reflect poor item design since it can create confusion for people who have difficulty with some elements of the item and not others; and can create a lack of discriminations if there are functional subgroups that manifest differently in that item.

Seventeen items of the RA-WIS could not be coded (“nc”) as the ICF did not have codes that were directly linked to the meaningful concepts. ICF linking does not incorporate coding related to personal factors and quality of life. The items that were “not codable” were mostly pertained to personal factors, for example, quitting work, worried about work, and good or bad days at work, which have more of an emotional component attached to them.

The IPF was included because it classifies the decision that the respondent will have to make to respond to an item (rational/emotional). The IPF was able to classify all items in all three questionnaires. The developer has provided a means of linking the IPF codes to ICF, in this study it augmented classifying items with ICF which could not have been done initially with the ICF alone. However, IPF has only recently been developed, and its role in the evaluation of content validity is not yet clear. Hence more studies are needed to see if the use of IPF to augment the ICF provides a useful method for assessing content.

The radar plot revealed that there was an overlap in the content of the three questionnaires with the ICF coding, with more items falling within the activity and participation domain and very minimal distribution across the other domains. However when the radar plot for the IPF classification was done, it showed that the questionnaires were quite different in the perspective which these domains were extracted from respondents, especially the SPS-6 which takes more of an emotional perspective. The vocational rehabilitation core set developed through international consensus may serve as a benchmark for the important concepts in vocational rehabilitation. We assessed alignment with the core set as being the extent to which items on the scale assessed concepts and scope of coverage as the percentage of core set codes addressed by the measure. A brief measure like the SPS-6 might be very well aligned but address a small scope of the core set. However, the SPS-6 was the measure that was best aligned and had the best scope. Our results show that, on an average, only 12% of the codes in the vocational rehabilitation core set were used in coding the presenteeism questionnaires. This suggests that none of the questionnaires we evaluated would be sufficient for assessment in vocational rehabilitation, since only 20 percent of total codes were from the vocational rehabilitation core set. For example, in a review of the core set, there were a few items of the questionnaires that are not amenable to self-report such as stating good or bad days at work clearly without defining the criteria for those good or bad days. Some concepts that are deemed important by the ICF were missing in the questionnaires.

This study informs our understanding of the content of these three questionnaires but has limitations. The use of only two independent coders may have affected the interpretation of coding although there was high consistency between raters. The concepts that were difficult to code because they contain content that was not assessed by ICF may have been approached differently by different raters. The strengths of the study are the use of IPF to augment and enhance the utility of ICF and the use of radar plots to represent diagrammatically where the meaningful concepts of the questionnaire items fall within the domains of ICF.

Implications for clinical practice, research and policy are as follows. This study has compared the content and perspective of these three presenteeism questionnaires. This may help researchers and clinicians to decide as to which outcome measure to select to quantify a particular end point. This could be of great help in conducting international multi-center clinical trials by facilitating the production of data that is comparable across borders and languages. This study may inform policy by providing outcome measures that can produce comparable and reliable data to provide population estimates of presenteeism. This can aid in planning disability and sick leave benefits.

## 5. Conclusion

Our study concluded that the three presenteeism questionnaires vary considerably in the content they address, in their relationship to the vocational rehabilitation core set, and in the proportion of content that could not be classified by the ICF. The IPF provided a different perspective on items. In particular, many of the items and codes such as the emotional could not be classified by the ICF, which does not deal with how people feel about specific constructs. The IPF illustrates a preponderance of focus on the social domain. We recommend further studies to look into the content of these presenteeism questionnaires and how they link to the vocational rehabilitation core set of the ICF.

## Figures and Tables

**Table 1:** Meaningful concepts and related IPF and ICF codes for the WLQ-26.

	Item	Meaningful concept	ICF code	IPF code
1	Get to work on time	Work on time	d 2301, managing daily routine d 840–859, work and employment	R_S
2	Stick to a routine or schedule without having to rearrange your work tasks	Stick every day to a particular schedule at work	d 230, carrying out daily routine d 840–859, work and employment	R_S
3	Work without taking frequent rests or breaks to avoid discomfort	Discomfort bothering work	b 289, pain specified and unspecified d 840–859, work and employment	R_S R_B
4	Work the required number of hours	Work throughout the specified hours of work	d 2302, completing the daily routine d 840–859, work and employment	R_S
5	Handle very demanding or stressful work situations	Handling stress and job demands at work	d 2401, handling stress d 840–859, work and employment	E_S E_P
6	Do your work without becoming tense or frustrated	Frustration or tension	b 1521, regulation of emotion d 840–859, work and employment	R_S R_P
7	Do your work carefully	Work carefully	b 1400, sustaining attention d 840–859, work and employment	R_S
8	Satisfy those people who judge your work	Satisfying people whom you work under by your work	e 430, individual attitudes of people in position of authority d 7101, appreciation in relationships d 840–859, work and employment	R_S
9	Feel a sense of accomplishment	Not clear whether satisfaction or completion at work	ND d 840–859, work and employment	E_S
10	Finish work on time	Complete work within the specified time period effectively	d 2301, managing daily routine d 840–859, work and employment	R_S
11	Handle the workload	Carrying out daily routine and handling responsibilities	d 230, carrying out daily routine d 2400, handling responsibilities d 840–859, work and employment	R_S
12	Lift, carry, or move objects at work weighing 10 pounds or less	Lifting and carrying objects using hands at work	d 430, lifting and carrying objects d 445, hand and arm use d 840–859, work and employment	R_S R_I
13	Lift, carry, or move objects at work weighing 10 pounds or more	Lifting and carrying objects using hands at work	d 430, lifting and carrying objects d 445, hand and arm use d 840–859, work and employment	R_S R_I
14	Walk more than one block or climb up or down one flight of stairs while working	Walking and climbing stairs during work	d 450, walking d 4551, climbing d 840–859, work and employment	R_S R_I
15	Sit, stand, or stay in one position for longer than 15 minutes while working	Ability to maintain body position at work	d 4153, maintaining a sitting position d 4154, maintaining a standing position d 415, maintaining a position d 840–859, work and employment	R_S R_B
16	Bend, twist, or reach while working	Bending, twisting, and reaching objects during work	d 4105, bending d 410, changing body position d 4452, reaching d 840–859, work and employment	R_S R_B
17	Use hand operated tools or equipment (e.g., pen, drill, sander, keyboard, or computer mouse)	Use tools related to work with hands	d 4453, turning or twisting the arms e 1350, general products and technology for employment d 840–859, work and employment	R_S R_I
18	Use your upper body to operate tools or equipment (upper body means arms, head, neck, shoulders, or upper back)	Use tools related to work with the upper body	d 4453, turning or twisting the arms e 1350, general products and technology for employment d 840–859, work and employment	R_S R_I
19	Use your lower body to operate tools or equipment (lower body means legs, knees, feet, or lower back)	Moving objects with lower extremities	d 435, moving objects with lower extremities d 840–859, work and employment	R_S R_I
20	Keep your mind on your work	Concentrating on work	b 140, sustaining attention d 840–859, work and employment	R_S R_P
21	Keep track of more than one task or project at the same time	Handling multiple task demands in work	d 2200, carrying out multiple tasks d 840–859, work and employment	R_S R_P
22	Concentrate on your work	Concentrating on work	b 140, sustaining attention d 840–859, work and employment	R_S R_P
23	Remember things having to do with your work	Remembering things at work	b 144, memory functions d 840–859, work and employment	R_S R_P
24	Talk with people in person, in meetings, or on the phone	Talking with a person or on the phone or using an operating device for work	d 350–d 369, conversation and use of communicative devices and techniques d 840–859, work and employment	R_S
25	Control irritability or anger toward people when working	Maintaining a healthy relationship with colleagues	b 1263, psychic stability d 840–859, work and employment	E_S
26	Help other people get work done	Helping others to do their work to get things done	d 6608, assisting others, others specified d 840–859, work and employment	R_S

d 840–859, work and employment used for all the items, b: body function, s: body structures, d: activity and participation, e: environmental factors.

R_S: rational appraisal social domain, R_B: rational appraisal biological domain, R_P: rational appraisal psychological domain, R_I: rational appraisal inorganic domain, E_S: emotional appraisal social domain, and E_P: emotional appraisal psychological domain.

**Table 2:** Table showing meaningful concepts and related IPF and ICF codes for the SPS-6.

	Item	Meaningful concept	ICF code	IPF code
1	Because of my depression, stress or anxiety, the stress of my job was much harder to handle	Difficulty in coping between stressors at work and due to depression	d 240, handling stress and other psychological demands d 840–859, work and employment	E_S E_P
2	Despite having my health problem, I was able to finish hard tasks in my work	Coped up well between health and every day job demands	d 240, handling stress and other psychological demands d 2301, managing daily routine d 840–859, work and employment	E_S E_B
3	My depression, stress, or anxiety distracted me from taking pleasure in my work	Stresses drifted the ease of enjoying work	b 152, emotional function b 1521, regulation of emotion d 840–859, work and employment	E_S E_P
4	I felt hopeless about finishing certain work tasks due to my health problem	Difficulty in completing certain work related tasks because of health issues	b 1521, regulation of emotion d 840–859, work and employment	E_S E_B E_P
5	At work I was able to focus on achieving my goals despite my health problem	Balanced well with achieving targets at work in spite of the health issue	d 2401, handling stress b 130, energy and drive functions d 840–859, work and employment	R_S R_B
6	Despite having my health problem I felt energetic enough to complete all my work	Completion of work with ease in spite of the health problem	b 152, emotional function b 1300, energy level d 2303, managing one's own activity level d 840–859, work and employment	R_S R_B

d 840–859: work and employment used for all the items, b: body function, s: body structures, d: activity and participation, e: environmental factors, R_S: rational appraisal social domain, R_B: rational appraisal biological domain, E_S: emotional appraisal social domain, E_P: emotional appraisal psychological domain, and E_B: emotional appraisal biological domain.

**Table 3:** Meaningful concepts and related IPF and ICF codes for the RA-WIS.

	Item	Meaningful concept	ICF code	IPF Code
1	I can get my job done; I am just a lot slower	Able to complete work but very slowly	d 850, remunerative employment NC d 840–859, work and employment	R_S
2	If I do not reduce my hours I may have to give up work	Reducing work hours Quit work	d 850, remunerative employment NC d 840–859, work and employment	R_S
3	I am very worried about my ability to keep working	Work Worried about continuing work	d 850, remunerative employment NC d 840–859, work and employment	E_S
4	I have pain or stiffness all the time at work	Work Pain or stiffness bothering work	d 850, remunerative employment b 280, pain sensation b 780, stiffness related to movement d 840–859, work and employment	R_S R_B
5	I do not have the stamina to work like I used to	Work Stamina to sustain work Stamina to sustain work like before	d 850, remunerative employment b 4550, stamina endurance to work NC d 840–859, work and employment	R_S R_B
6	I have used my holiday so that I do not have to go on sick leave	Used up holidays Used up holidays so will not go on sick leave	d 850, remunerative employment NC d 840–859, work and employment	R_S
7	I push myself to go to work because I do not want to give into my shoulder/elbow problem	Motivate myself to work so that I do not rest on to my shoulder or elbow problem	d 850, remunerative employment b 1301, motivation NC d 840–859, work and employment	E_S
8	Sometimes I cannot face being at work all day	Work Face being at work	d 850, remunerative employment NC d 840–859, work and employment	R_S
9	I have to say no to certain things at work	Work and employment Say no to certain things not very clear what they really mean	d 850, remunerative employment NC d 840–859, work and employment	R_S
10	I have got to watch how much I do certain things at work	Work Watch on certain things at work	d 850, remunerative employment NC d 840–859, work and employment	R_S
11	I have great difficulty opening some of the doors at work	Work and employment Does not explain either physically or mentally unable	d 850, remunerative employment NC d 840–859, work and employment	R_S R_I
12	I have to allow myself extra time to do some jobs	Work and employment Allocating extra time for other jobs	d 850, remunerative employment NC d 840–859, work and employment	R_S
13	It is very frustrating because I cannot always do things at work	Work and employment Frustration cannot do things at work	d 850, remunerative employment NC d 840–859, work and employment	E_S
14	I feel I may have to give up work	Work and employment Giving up work	d 850, remunerative employment NC d 840–859, work and employment	E_S
15	I get on with work but afterwards I have a lot of pain	Work and employment Pain Pain after doing activity	d 850, remunerative employment b 280 NC d 840–859, work and employment	R_B
16	When I am feeling tired all the time work is a grind	Work and employment Work is a grind	d 850, remunerative employment b 4552, fatigability NC d 840–859, work and employment	R_S R_P
17	I would like another job but I am restricted to what I can do	Work and employment Restricted to doing the job despite being interested in another job	d 850, remunerative employment d 845, acquiring, keeping, and terminating a job d 840–859, work and employment	R_S
18	I get up earlier because of my shoulder/elbow problem	Work and employment Waking up early because of pain	d 850, remunerative employment NC d 840–859, work and employment	R_S R_B
19	I get very stiff at work	Work and employment Stiffness	d 850, remunerative employment b 780, stiffness d 840–859, work and employment	R_S R_B
20	I am finding my job is all about all I can manage	Work and employment Managing work	d 850, remunerative employment d 230, carrying out daily routine d 840–859, work and employment	R_S
21	The stress of my job makes my shoulder/elbow problem flare	Work and employment Stress in job makes elbow or shoulder problem worsen	d 850, remunerative employment d 2401, handling stress NC d 840–859, work and employment	E_S E_B
22	Any pressure on my hands is a problem	Work and employment Pressure on hands	d 850, remunerative employment b 2702, sensitivity to pressure d 840–859, work and employment	R_B
23	I have good days and bad days at work	Work and employment Good and bad days at work	d 850, remunerative employment NC d 840–859, work and employment	E_S

d 840–859: work and employment used for all the items, d 840–859: Work and employment used for all the items, b: body function, s: body structures, d: activity and participation, and e: environmental factors.

R_S: rational appraisal social domain, R_B: rational appraisal biological domain, R_P: rational appraisal psychological domain, R_I: rational appraisal inorganic domain, E_S: emotional appraisal social domain, E_P: emotional appraisal psychological domain, and E_B: emotional appraisal biological domain.

**Table 4:** Scope and alignment of the questionnaires when compared to the ICF core set for vocational rehabilitation.

Questionnaire (total number of items)	Alignment*		Scope	
	Brief core set	Comprehensive core set	Brief core set	Comprehensive core set
WLQ-26 (26)	7%	88%	28.6%	11.4%
SPS-6 (6)	83%	100%	54.5%	18.2%
RA-WIS (23)	9%	30%	22.9%	11.4%

*Does not account for the use of mandatory work related codes (d 840–859). Else with the mandatory work related codes being used for all the items the alignment would be perfect (100%).

WLQ-26: the 26-itemWork Limitation Questionnaire; SPS-6: the Stanford Presenteeism Scale-6; RA-WIS: Rheumatoid Arthritis Work Instability Scale. Alignment: percentage of the items that go to core set. Scope: the percentage of codes from core set reflected on the measure.
